# Moving-Principal-Component-Analysis-Based Structural Damage Detection for Highway Bridges in Operational Environments

**DOI:** 10.3390/s24020383

**Published:** 2024-01-08

**Authors:** Ye Yuan, Xinqun Zhu, Jun Li

**Affiliations:** School of Civil and Environmental Engineering, University of Technology Sydney, Ultimo, NSW 2007, Australia; ye.yuan@student.uts.edu.au (Y.Y.); jun.li-2@uts.edu.au (J.L.)

**Keywords:** moving principal component analysis, structural damage detection, moving vehicles

## Abstract

With the deterioration of bridge performance and ever-increasing amounts of traffic, bridge safety is becoming a concern for the engineering community. A method that can assess a bridge’s condition in real time is urgently needed. The main factors that hinder bridge condition assessment are the uncertain operational environments. A new moving principal component analysis (MPCA)-based method is developed for structural damage detection in bridges in operational environments in this paper. Two main operational environmental factors, the environmental temperature and traffic loads, are studied in the assessment process to verify the robustness and practicality of the proposed method. The numerical and experimental results show that the proposed method is effective and accurate in assessing the bridge’s condition in operational environments.

## 1. Introduction

Bridges are key components of transportation infrastructure that are crucial for a society to function well. They are under increasing pressure from continuing deterioration due to ageing and operational and environmental loading as a result of population growth and climate change. The real-time assessment of a bridge’s status is urgently required for structural safety. Structural health monitoring (SHM) provides a practical tool to assess and predict the structural performance of bridges. SHM is a multi-disciplinary field involving data collection using sensor networks and the diagnosis of structural health based on the collected data [1]. The collected data are processed to extract features that can be analysed through model-based or data-driven techniques to enhance decision making for structural condition assessment [2,3,4]. The key factors that hinder a reliable bridge condition assessment are the various operational and environmental factors, especially traffic excitation and environmental temperature [5]. Bridge condition assessment under operational environments has drawn enormous attention from researchers and practical engineers [6].

There are two main research trends used to deal with uncertain operational environments, e.g., elimination and utilisation. Different methods have been developed to separate the temperature-induced response from the structural response and to predict the temperature-induced response [7]. Modal parameters are widely used for structural damage detection. The change in modal parameters due to temperature variations is differentiated and eliminated during the process of damage detection [8,9]. An artificial neural network model for the temperature-induced response is trained using a large amount of data, and the model is used to compensate for the temperature effect [10,11]. The Kalman filter is adopted to eliminate the change due to environmental variations [12]. A two-stage procedure based on adaptive Mahalanobis-squared distance and one-class K-nearest neighbours (KNN) is used to remove the environmental variability [13]. A long period of monitoring data (e.g., for months) is used to construct the prediction model of structural response under operational environments for structural damage detection [14,15]. The above data-driven methods under changing temperature conditions reveal great potential for practical use with high robustness. These methods can identify, eliminate, or utilise the influence of the environmental temperature on the bridge’s response. The biggest obstacle to the performance of these methods comes from the availability of the data for all possible operational environments in bridge health monitoring. The performance of the regression models or neural networks has been affected by high-quality, long-term monitoring data.

The utilisation, known as the ambient vibration survey (AVS), aims to use dynamic responses under natural excitations, such as traffic loads, wind, and micro-tremors [16]. The AVS is an economical, convenient, and time-saving approach as it does not require a special excitation on structures. For bridge engineering, the vehicle–bridge interaction is a common problem that will affect the accuracy of dynamic analysis. A great effort has been made to fully understand its mechanism for practical applications [17]. Based on these studies, the bridge information could be extracted from the traffic-induced response using the vehicle–bridge interaction model. Recently, moving-load-driven bridge damage detection has drawn much attention. The moving vehicle could be an exciter and a moving sensor for real-time monitoring of the bridge [18,19]. The duration of a vehicle passing the bridge is short, and the change due to the temperature effect is relatively small within this short time period. The traditional moving-load-driven methods have good interpretability, as these methods are based on the accurate vehicle–bridge interaction model [20]. These methods are suitable for controlled environmental conditions, but it is laborious and time consuming to ensure their performances due to their limited anti-noise ability [21].

The principal component analysis (PCA) is one of most widely used data-driven methods. It is based on an orthogonal decomposition of the covariance matrix of the process variables along the direction that explains the maximum variation in the data. The PCA has been used to separate the structural damage features from those of the environmental changes, and then the environmental effects are eliminated during the process of bridge condition assessment [22]. The PCA has been used to reduce the size of feature vectors [23], eliminate the operational and environmental effects [24,25], and reduce the noise effect [26] for structural damage detection. With the traditional PCA, the whole time series of measured responses is analysed, and it cannot reflect the instant status of the structure [27]. Also, the computational cost of the covariance matrix will be increased with the number of measurements and the length of the time series [28]. Posenato et al. [29] proposed a data-driven method named moving principal component analysis (MPCA) for long-term structural monitoring. MPCA calculates the covariance matrix in a fixed-length window. Lanata et al. [28] used MPCA to capture the correlation in each small cluster obtained using the K-means method for structural damage detection. Cavadas et al. [30] compared the performance of structural damage detection using MPCA and robust regression analysis (RRA). Zhu et al. [31] proposed a temperature-driven damage detection approach for bridges considering the temperature variations and traffic loads using MPCA. Jin et al. [32] compared the performance of the modal-analysis-based damage detection method under changing temperatures using PCA or MPCA. The results revealed that MPCA has more anti-noise ability with lower false alarm rates than PCA. Zhang et al. [33] deployed MPCA for damage detection on a rigid frame bridge under seasonal temperature variations with space and time windows. Jin and Jung [34] compared modified MPCA with the static linear principal component analysis (SPCA) and incremental linear principal component analysis (IPCA) on a Z24 bridge dataset using k-means clustering with the Linde–Buzo–Gray algorithm (KMC-LBG) and Bayesian information criterion (BIC) to choose the window size of MPCA. Nie et al. [27] developed a narrow moving window for MPCA and successfully detected the real-time change of an actual suspension bridge. The window’s size is decided based on the cumulative contribution ratio with a convergent spectrum. As stated above, MPCA has a great potential for early warning and damage detection as it can reveal the data’s inherent correlation and structure in detail. The length of the moving window significantly affects the effectiveness of the MPCA-based method. How to select the appropriate window length is still a big challenge. The loading forms and the causes of bridge anomalies (e.g., damage) are often inconsistent. This inconsistency means the results of MPCA are not interpretable.

This paper aims to develop a new method for bridge condition assessment under operational environments. A new damage-sensitive feature (DSF) based on moving principal component analysis (MPCA) has been proposed. Two main environmental factors, e.g., the temperature variations and traffic loads, are considered. The numerical and experimental studies have been conducted to verify the performance of the proposed method. In the numerical study, the vehicle–bridge interaction system is modelled as a simply supported Euler–Bernoulli concrete beam subjected to a moving load. The bridge damage is simulated using a breathing crack. The temperature influence is considered as the variation in the beam’s parameters and the thermal stress induced by the vertical temperature difference. The results show that the vehicle’s mass and the temperature do not have an effect on the accuracy of structural damage identification. The changing pattern of the proposed DSF reflects different damage locations. The experimental study of a T-section reinforced concrete beam subjected to moving vehicles with different weights has been conducted in the laboratory. A new type of window for MPCA is proposed to filter out the effect of measurement noise and vehicle–bridge interaction. The results show that the proposed method is robust and accurate in detecting the crack damage of the bridge under operational environments.

## 2. Theory

PCA and MPCA are briefly introduced in this section. Detailed information can be found in the textbook [35]. Then, the main target for current, existing data processing algorithms is discussed.

### 2.1. Principal Component Analysis (PCA)

As the foundation of MPCA, PCA is a statistical learning method that decomposes the original data into linearly uncorrelated vectors, principal components (PCs), according to the maximum variance’s direction. At the same time, the new coordinate axis’s direction should be orthogonal to all previous coordinate axes’ directions. This transformation ensures that the selection of the coordinate axis’s direction can make each PC contain as much information as possible. It is widely used in data compression and feature extraction.

In this study, the singular value decomposition (SVD) method is adopted for PCA. Considering a data (signal) matrix $X_{m\times n}$ with the k-order PCs, suppose the matrix’s rank is *r* (that is, greater than or equal to *ki*). The matrix $X_{m\times n}$ can be factorised according to truncated SVD as

(1) $$X_{m\times n}\approx U_{k}\Sigma_{k}V_{k}^{T}$$

where $U_{k}$ is the m×k matrix, $V_{k}$ is the n×k matrix, and $\Sigma_{k}$ is the diagonal matrix of the order k. $U_{k}$ and $V_{k}$ are, respectively, taken from the first k columns of matrices U,V**,** which are singular vectors of the matrix $X_{m\times n}$. $\Sigma_{k}$ is obtained from the first k diagonal elements of matrix Σ**,** which is the singular value matrix of the matrix $X_{m\times n}$.

Before using PCA, the data in the matrix $X_{m\times n}$ need to be standardised to obtain the matrix X’ as

(2) $$x_{ij}^{*}=\frac{x_{ij}-\bar{x_{i}}}{\sqrt{s_{ii}}}$$

where $\bar{x_{i}}=\frac{1}{n}\sum_{j=1}^{n}x_{ij},i=1,2,...,m$ and $s_{ii}=\frac{1}{n-1}\sum_{j=1}^{n}(x_{ij}-\bar{x_{i}}{)}^{2},i=1,2,...,m$. $x_{ij}$ is the $X_{m\times n}$’s element at row i and column j. $x_{ij}^{*}$ is the standardised matrix X’’s element at row i and column j. $\bar{x_{i}}$ is the average value in row i of the matrix X’.

After obtaining the standardised data matrix X’, the traditional PCA uses the eigenvalue decomposition of the X’’s correlation matrix or covariance matrix to calculate the principal component matrix. According to the characteristic of SVD, the principal component matrix can be obtained:

(3) $$X^{\prime \prime }=\frac{1}{\sqrt{n-1}}{X’}^{T}$$

(4) $$X^{\prime \prime }=U\Sigma V^{T}$$

(5) $$Y_{k\times n}=V^{T}X’$$

where $X^{\prime \prime }$ is constructed for the truncated SVD. The row of $V^{T}$ is the eigenvector of the X′’s covariance matrix. $Y_{k\times n}$ is the principal component or score matrix. $V^{T}$ is the weight or coefficient matrix.

### 2.2. Moving Principal Component Analysis (MPCA)

**Figure 1:**
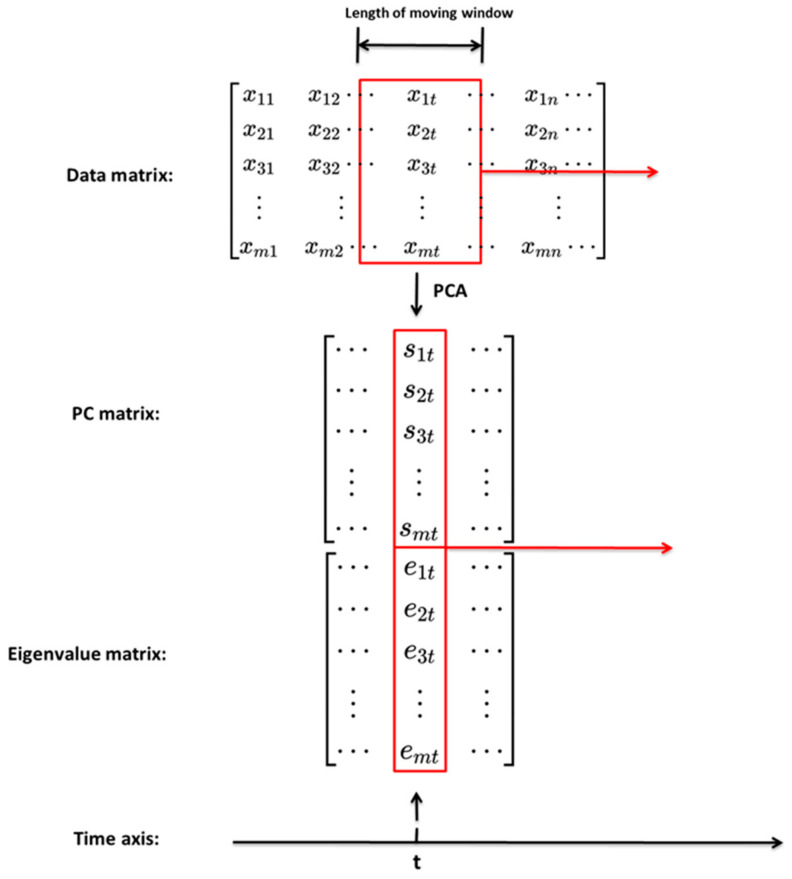
The schema of MPCA.

For time series signals, the analysis within a sliding window can better excavate the features of the data. MPCA is a method that deploys PCA on the signal truncated into the window length instead of the full signal. The window is like a filter that slides and decomposes the original signals along the time axis to different PCs. These PCs obtained from MPCA have more significant features than the PCs obtained from PCA. Likewise, the window expands the eigenvalues into a series of changing curves along the time axis. These curves can better reflect the inner variations of the data over time. The main difference between MPCA and PCA is that Equation (1) in MPCA is calculated over the signal of the window length, not over the entire signal. When the window moves from the initial time to the end, the eigenvalue vector and the PCs are calculated simultaneously at each corresponding time t and saved consecutively in the eigenvalue matrix and the principal component matrix, respectively, according to the order of the time axis. The coordinate of the eigenvalue vector and the PCs obtained at each time t from Equations (4) and (5) corresponds to the location of the window’s centre. The window moves along the time axis step by step from beginning to end. In each movement PCA is performed on the data segments intercepted within the window. The data obtained by performing PCA at each movement only retains the single data point at the centre of the window. This data point is stored on the time axis corresponding to the position of the window coordinates. These data points obtained by each window movement are concatenated one by one on the time axis to form the eigenvalue vectors and PCs. Figure 1 shows the schema of MPCA.

## 3. Numerical Study

In this section, the vehicle–bridge interaction model with the temperature change and the crack damage is first built. Dynamic responses of the bridge with different damage scenarios subject to a moving vehicle are simulated. The dynamic responses are analysed using PCA and MPCA for comparison. Then, the damage-sensitive feature is constructed based on the analysis. The influence of the crack’s location and the breathing crack mechanism on the damage-sensitive feature is discussed.

### 3.1. Vehicle–Bridge Interaction Model

#### 3.1.1. Finite Element Model of a Beam Bridge

The bridge is discretised as N−1 beam elements, and N is the number of nodes. The element mass matrix and stiffness matrix of a beam element can be obtained as

(6) $$M_{e}=\frac{\rho Al}{420}\left[\begin{array}{cccc} 156 & 22l & 54 & -13l\\ 22l & 4l^{2} & 13l & -3l^{2}\\ 54 & 13l & 156 & -22l\\ -13l & -3l^{2} & -22l & 4l^{2} \end{array}\right]\;\;K_{e}=\frac{EI}{l^{3}}\left[\begin{array}{cccc} 12 & 6l & -12 & 6l\\ 6l & 4l^{2} & -6l & 2l^{2}\\ -12 & -6l & 12 & -6l\\ 6l & 2l^{2} & -6l & 4l^{2} \end{array}\right]$$

where ρ,A,l are the density, the section’s area, and the length of the beam element, respectively.

**Figure 2:**
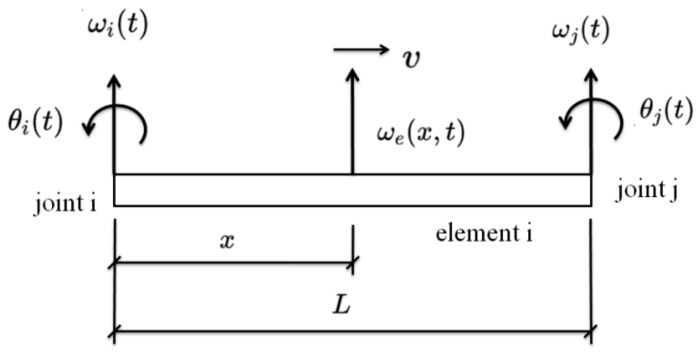
The Hermite interpolation on the beam element.

Figure 2 shows the *i*th beam element. The response at point x and time t can be obtained using the Hermite interpolation $H\left(x\right)$ from the node responses. The Hermite interpolation H(x) is as follows:

(7) $$\begin{array}{c} H_{1}\left(x\right)=1-3{\left(\frac{x}{l}\right)}^{2}+2{\left(\frac{x}{l}\right)}^{3}\\ H_{2}\left(x\right)=x{\left(1-\frac{x}{l}\right)}^{2}\\ H_{3}\left(x\right)=3{\left(\frac{x}{l}\right)}^{2}-2{\left(\frac{x}{l}\right)}^{3}\\ H_{4}\left(x\right)=\frac{x^{2}}{l}\left(\frac{x}{l}-1\right) \end{array}$$

The displacement at point x and time t is obtained as:

(8) $$\omega_{e}\left(x,t\right)={H(x)}^{T}R\left(t\right)=\left\{\begin{array}{cccc} H_{1}\left(x\right) & H_{2}\left(x\right) & H_{3}\left(x\right) & H_{4}\left(x\right) \end{array}\right\}\left\{\begin{array}{c} \omega_{i}\left(t\right)\\ \theta_{i}\left(t\right)\\ \omega_{j}\left(t\right)\\ \theta_{j}\left(t\right) \end{array}\right\}$$

where $\omega_{e}\left(x,t\right)$ is the displacement at point *x* and time t. $\omega_{i}\left(t\right),\;\theta_{i}\left(t\right)$ are the node displacement and rotation at the $i^{th}$ node and time t of the beam element. x is the position and l is the length of the beam element. $R\left(t\right)$ are the node responses.

The strain at point x and time t can also be obtained [5] as

(9) $$\varepsilon \left(x,t\right)=-z\frac{\begin{array}{c} \partial H(x)R\left(t\right) \end{array}}{\partial x^{2}}$$

where z is the distance from the bottom to the neutral axis.

#### 3.1.2. Equation to Calculate the Motion of the Bridge Subjected to a Moving Vehicle

**Figure 3:**
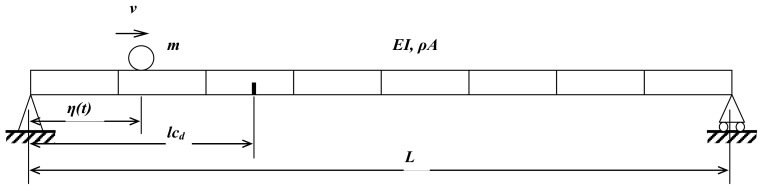
The damaged beam under a moving mass.

The bridge is modelled as a simply supported beam, and the vehicle is modelled as a mass m, as shown in Figure 3. The bridge length is L. The vehicle is moving along the bridge at a constant speed v. The crack damage is considered in this study. $lc_{d}$ is the crack’s location from the left support. The beam bridge is discretised into N−1 elements, and N is the number of nodes. Considering Rayleigh damping for the bridge, the motion of the bridge subjected to a moving vehicle can be obtained as follows:

(10) $$M_{b}\ddot{R}\left(t\right)+C_{b}\dot{R}\left(t\right)+K_{b}R\left(t\right)=H_{m}P$$

where $M_{b},C_{b}$, and $K_{b}$ are the mass, damping, and stiffness matrices of the bridge, respectively. The $\ddot{R}\left(t\right),\dot{R}\left(t\right)$, and $R\left(t\right)$ are the node’s acceleration, velocity, and displacement response vectors, respectively. $H_{m}P$ is the node’s equivalent force vector induced by the moving mass. P is the equivalent resultant force vector induced by the moving mass. The interaction force between the bridge and the mass is $P(x\left(t\right),t)$, which can be obtained as

(11) $$P\left(x\left(t\right),t\right)=m\left\{g-\frac{d^{2}\left(\omega \left(x\left(t\right),t\right)\right)}{dt^{2}}\right\}$$

where m is the mass of a moving vehicle. $H_{m}={\left\{\begin{array}{cccccc} 0 & \cdots & H(\eta (t){)}_{j}^{T} & 0 & \cdots & 0 \end{array}\right\}}^{T}$ when $\left(j-1\right)l\leq \eta (t)\leq jl$. H(x) is the Hermite interpolation as the beam element’s shape function. The beam’s deflection at point x and time t can be written as

(12) $$\omega \left(x,t\right)=H\left(x\right)R\left(t\right)$$

where $H(x)={\left\{\begin{array}{cccccc} 0 & \cdots & H(x{)}_{j}^{T} & 0 & \cdots & 0 \end{array}\right\}}^{T}$ when (j−1)l≤x≤jl. Combining Equations (10)–(12), the equation of motion can be written as

(13) $$M(t)\ddot{R}(t)+C(t)\dot{R}(t)+K(t)R(t)=mgH_{m}$$

where $M(t)=M_{b}+mH_{m}H(x)$, $C(t)=C_{b}+2mvH_{m}H’(x)$, and $K(t)=K_{b}+mv^{2}H_{m}H^{\prime \prime }(x)$. H’(x) and $H^{\prime \prime }(x)$ are the first and second derivatives of the Hermite interpolation vector H(x). Equation (13) can be solved using the Newmark-β method. The parameters are: α=0.5, β=0.25. The time step is 0.01 s. Then, the bridge response at point x can be obtained through Equations (7)–(9).

#### 3.1.3. Temperature Influence

The temperature’s influence can be divided into two parts. The first one is the variation in the beam’s parameters, which will directly influence the beam’s dynamic property. In the model, the thermal coefficients of the temperature’s influence on each parameter are listed in Table 1 [16]. $A_{0}$, $E_{0}$, and $I_{0}$ are the cross-section area, Young’s modulus, and second moment of inertia at the reference temperature $T_{0}$, respectively. The temperature effect on the section Young’s modulus depends on the load-bearing ratio of section’s each material. According to the load-bearing ratio at the reference temperature, the coefficients corresponding to the temperature effect of steel and concrete are linearly weighted according to this ratio to obtain the final temperature influence coefficient of the entire section. The section’s expansion only considers the concrete’s area growth.

The second influence is induced by the vertical temperature gradient. The bending curvature of the beam is affected by the temperature gradient. For a simply supported beam, the influence of the vertical temperature gradient can be taken as [36]:

(14) $$r_{1}=-\frac{\alpha l_{0}\cdot \Delta T}{2\sqrt{3}\cdot h}\cdot \frac{\lambda +1}{\lambda -1}$$

(15) $$r_{2}=-r_{1}$$

(16) $$M_{1}=\left[1-\frac{1}{\sqrt{3}}\cdot \frac{\lambda +1}{\lambda -1}\right]\cdot \frac{\alpha EI\cdot \Delta T}{h}$$

(17) $$M_{2}=M_{1}$$

where $r_{1}$ is the vertical temperature gradient induced rotation at the beam’s left support. $M_{1}$ is the vertical temperature gradient induced moment at the beam’s left support. $r_{2}$ is the vertical temperature gradient induced rotation at the beam’s right support. $M_{2}$ is the vertical temperature gradient induced moment at the beam’s right support. ΔT is the temperature difference between the top and bottom surfaces, and the positive value reflects that the top surface is warmer. h is the cross-section’s height. EI is the beam’s rigidity. α is the concrete’s thermal expansion coefficient in Table 1. λ is a constant, and it is equal to $\frac{\sqrt{3}+1}{\sqrt{3}-1}$. $l_{0}$ is the beam’s length. According to Equations (14)–(17), the effect of the vertical temperature gradient is considered the boundary condition of the beam.

#### 3.1.4. Crack Damage Model

The beam’s damage starts from initial microcracks and develops due to many factors, such as degradation, loads, temperature impact, etc. While expanding, these microcracks keep opening and closing due to external dynamic excitation. This phenomenon is known as the breathing crack, and it dominates the beam’s crack behaviour in the incipient crack stage [37]. Because the prestress is widely applied, the crack in the prestressed concrete bridges will perform as the breathing crack.

**Figure 4:**
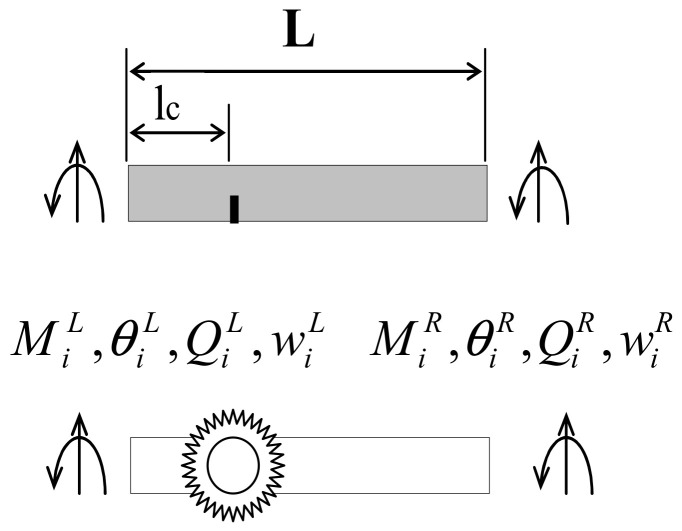
The cracked beam element.

In this study, the breathing crack is used to model the bridge damage [38]. The breathing crack is simulated as a rotational spring at the crack’s location $l_{c}$. Figure 4 shows the beam element with a breathing crack. This element is considered as two undamaged beam segments connected by the proposed rotational spring. EI is the undamaged beam’s flexural rigidity, and l is the length of this element. The stiffness matrix of this element can be written as

(18) $$\left\{\begin{array}{c} Q_{i}\\ M_{i}\\ Q_{d}^{L}\\ M_{d}^{L} \end{array}\right\}=\frac{EI}{l_{c}^{3}}\left[\begin{array}{cccc} 12 & 6l_{c} & -12 & {6l}_{c}\\ 6l_{c} & 4l_{c}^{2} & -6l_{c} & 2l_{c}^{2}\\ -12 & -6l_{c} & 12 & -6_{c}\\ 6l_{c} & 2l_{c}^{2} & -6l_{c} & 4l_{c}^{2} \end{array}\right]\left\{\begin{array}{c} w_{i}\\ \theta_{i}\\ w_{d}^{L}\\ \theta_{d}^{L} \end{array}\right\}$$

(19) $$\left\{\begin{array}{c} Q_{d}^{R}\\ M_{d}^{R}\\ Q_{j}\\ M_{j} \end{array}\right\}=\frac{EI}{{\left(l-l_{c}\right)}^{3}}\left[\begin{array}{cccc} 12 & 6(l-l_{c}) & -12 & 6(l-l_{c})\\ 6(l-l_{c}) & 4{\left(l-l_{c}\right)}^{2} & -6(l-l_{c}) & 2{\left(l-l_{c}\right)}^{2}\\ -12 & -6(l-l_{c}) & 12 & -6(l-l_{c})\\ 6(l-l_{c}) & 2{\left(l-l_{c}\right)}^{2} & -6(l-l_{c}) & 4{\left(l-l_{c}\right)}^{2} \end{array}\right]\left\{\begin{array}{c} w_{d}^{R}\\ \theta_{d}^{R}\\ w_{j}\\ \theta_{j} \end{array}\right\}$$

where $w_{i}$, $w_{j}$, $\theta_{i}$, and $\theta_{j}$ are the displacements and rotations at the *ith* and *jth* nodes, respectively. $Q_{i}$, $Q_{j}$, $M_{i}$, and $M_{j}$ are their corresponding transverse shear forces and moments. $w_{d}^{L}$, $\theta_{d}^{L}$, $w_{d}^{R}$, and $\theta_{d}^{R}$ are the spring’s displacements and rotations at the left- and right-hand sides of the joint, and $Q_{d}^{L}$, $M_{d}^{L}$, $Q_{d}^{R}$, and $M_{d}^{R}$ are their corresponding shear forces and moments.

**Figure 5:**
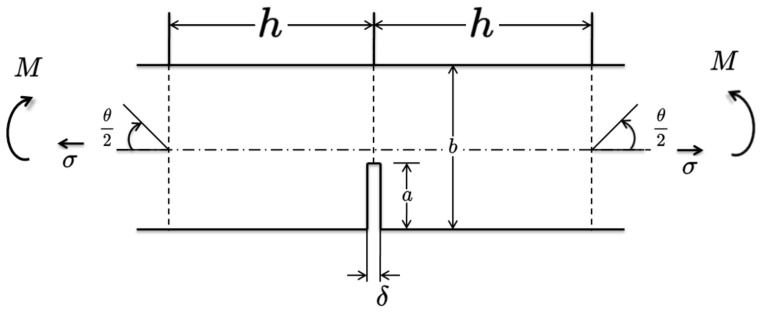
The crack opening at the edge.

According to the equilibrium and compatibility condition at the crack’s location, the cracked beam’s element stiffness matrix can be obtained.

(20) $$K_{d}=K_{1}+K_{2}K_{3}^{-1}K_{4}$$

where

$$K_{1}=\frac{EI}{l^{3}}\left[\begin{array}{cccc} \frac{12}{\delta^{3}} & \frac{6l}{\delta^{2}} & 0 & 0\\ \frac{6l}{\delta^{2}} & \frac{4l^{2}}{\delta } & 0 & 0\\ 0 & 0 & \frac{12}{(1-\delta {)}^{3}} & -\frac{6l}{(1-\delta {)}^{2}}\\ 0 & 0 & -\frac{6l}{(1-\delta {)}^{2}} & \frac{4l^{2}}{\left(1-\delta \right)} \end{array}\right]K_{2}=\frac{EI}{l^{3}}\left[\begin{array}{cccc} -\frac{12}{\delta^{3}} & \frac{6l}{\delta^{2}} & 0 & 0\\ -\frac{6l}{\delta^{2}} & \frac{2l^{2}}{\delta } & 0 & 0\\ 0 & 0 & -\frac{12}{(1-\delta {)}^{3}} & -\frac{6l}{(1-\delta {)}^{2}}\\ 0 & 0 & \frac{6l}{(1-\delta {)}^{2}} & \frac{2l^{2}}{\left(1-\delta \right)} \end{array}\right]$$

$$K_{3}=\left[\begin{array}{cccc} -\frac{12}{\delta^{3}} & \frac{6l}{\delta^{2}} & 0 & 0\\ \frac{6l}{\delta^{2}} & S-\frac{4l^{2}}{\delta } & 0 & 0\\ 0 & 0 & \frac{12}{(1-\delta {)}^{3}} & \frac{6l}{(1-\delta {)}^{2}}\\ 0 & -S & -\frac{6l}{(1-\delta {)}^{2}} & S-\frac{4l^{2}}{\left(1-\delta \right)} \end{array}\right]K_{4}=\left[\begin{array}{cccc} -\frac{12}{\delta^{3}} & -\frac{6l}{\delta^{2}} & 0 & 0\\ \frac{6l}{\delta^{2}} & \frac{2l^{2}}{\delta } & 0 & 0\\ 0 & 0 & -\frac{12}{(1-\delta {)}^{3}} & \frac{6l}{(1-\delta {)}^{2}}\\ 0 & 0 & -\frac{6l}{(1-\delta {)}^{2}} & \frac{2l^{2}}{\left(1-\delta \right)} \end{array}\right]$$

$$\delta =\frac{l_{c}}{l},S=\frac{K_{rd}l^{3}}{EI}$$

where $K_{rd}$ is the tangent stiffness, which reveals the spring’s instant rigidity. As shown in Figure 5, there is a crack opening at the edge of a rectangular section, and 2h is the cracked element’s length, while b is the element’s height. The crack is started at the centre of the element’s long edge. The rotational displacement due to the crack opening at the edge can be obtained using linear–elastic fracture mechanics, as follows [39]:

(21) $$\theta_{crack}=\frac{4\sigma }{E}S\left(\frac{a}{b}\right)$$

where σ is the applied stress of the whole cracked element induced by the bending moment. The $\frac{a}{b}$ is the ratio of the crack’s depth. For $\frac{h}{b}>2$, the $S(\frac{a}{b})$ can be written as [39]

(22) $$S\left(\frac{a}{b}\right)={\left(\frac{\frac{a}{b}}{1-\frac{a}{b}}\right)}^{2}\left\{5.93-19.69\left(\frac{a}{b}\right)+37.14{\left(\frac{a}{b}\right)}^{2}-35.84{\left(\frac{a}{b}\right)}^{3}+13.12{\left(\frac{a}{b}\right)}^{4}\right\}$$

The relationship of σ and M for the pure bending element can be written as

(23) $$\;\sigma =\frac{6M}{b^{2}}$$

where M is the cracked element’s bending moment. b is the length of the section’s short edge. Combining Equations (21)–(23), the instant tangent stiffness of the virtual rotational spring $K_{rd}$ is obtained.

(24) $$K_{rd}=\frac{M}{\theta_{crack}}=\frac{Eb^{2}}{24S(\frac{a}{b})}$$

### 3.2. Results and Discussions

#### 3.2.1. Numerical Simulation

The numerical model is validated through comparison with the results of Zhu and Law [40]. A simply supported beam that is 50 m long, 0.5 m wide, and 1 m high is used. The elastic modulus of the beam is $2.1\times 10^{11}$ Pa, and the density is $7860\;kg/m^{3}$. The moving force is 10 kN. The first six natural frequencies of the beam are listed in Table 2.

**Figure 6:**
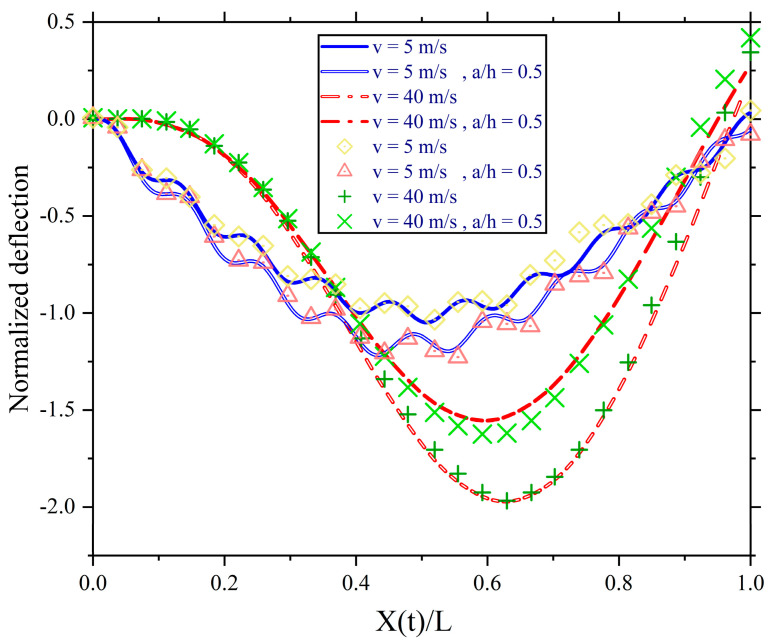
The normalised deflections at mid-span (scattered points obtained by Zhu and Law [40] and line curves obtained using the proposed method).

Figure 6 shows the normalised deflection at the mid-span. The sampling rate is 100 Hz. The deflection is normalised by $\frac{F_{0}L^{3}}{48EI}$, which is the static deflection when the force is at the mid-span. The scattered points are the analytical solution obtained by Zhu and Law [40], and the line curves are the numerical solution obtained using the proposed model in this study. a/h is the crack depth ratio at the mid-span. For v = 5 m/s, the number of elements is 13. For v = 40 m/s, the number of elements is 7. The number of elements is consistent with the compatibility condition in Equation (22). The results obtained using the proposed model are close to those of Zhu and Law [40]. This validated model will be used in this study.

#### 3.2.2. Comparison of Results Obtained Using PCA and MPCA

A comparison study is conducted in this section. The moving load is 10 kN. The velocity of the moving load is 5 m/s, and the number of elements is 13. Other parameters are the same as those in Section 3.2.1. The beam is intact. A sudden and slight change in the moving mass is simulated to illustrate the sensitivity and reliability of MPCA. The acceleration responses are analysed through PCA and MPCA. As listed in Table 3, three cases are studied.

**Figure 7:**
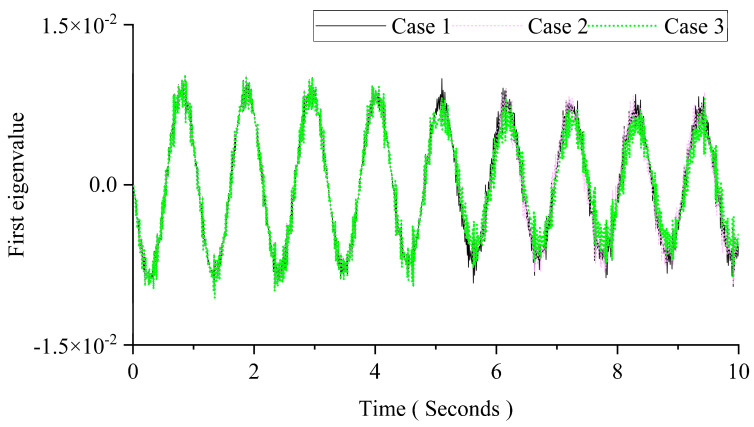
Results of PCA with three different masses.

Figure 7 shows the first PC for those three cases using PCA. There are no obvious changes in those cases in Figure 7. The results show that the PCA could not indicate the changes in the moving mass. The instantaneous state of the vehicle–bridge interaction system cannot be captured by conducting PCA on the whole time series.

**Figure 8:**
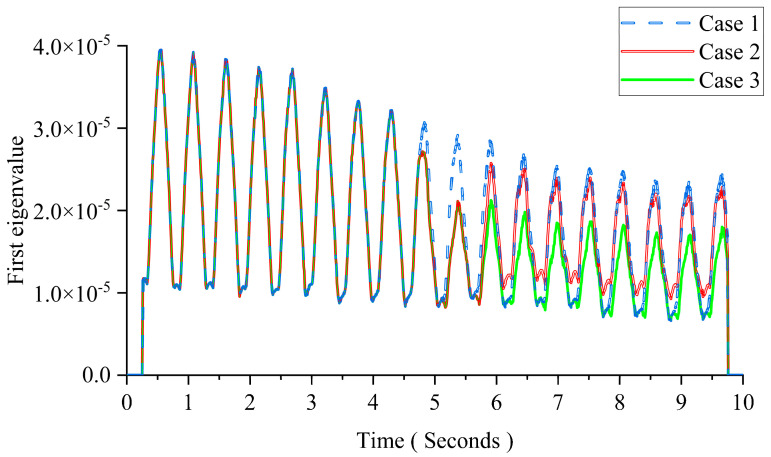
Result of MPCA with the mass change.

The eigenvalues are more sensitive to abrupt changes than the PCs [27]. The first eigenvalue is taken for comparison in this study. The size of the window is 50 times the sampling interval. Figure 8 shows the result using MPCA. From Figure 8, there are clear changes at 5 s in Cases 2 and 3. The results show that the 1% mass variation can be detected immediately in the first eigenvalue curve using MPCA. For Case 2, the magnitude of the first eigenvalue is the same as Case 1 after 6 s as the mass returns back to the original value. For Case 3, the first eigenvalue keeps approximately the same magnitude after 5 s because the mass of the moving load does not change after that.

#### 3.2.3. The Effect of Damage Patterns

**Figure 9:**
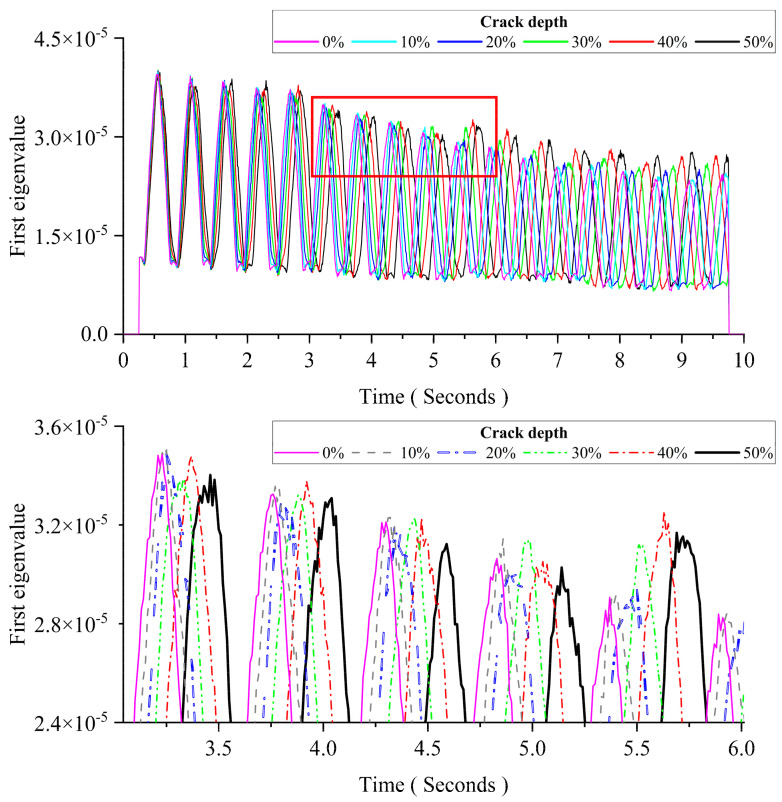
The influence of the crack depth for MPCA.

This section aims to study the effect of the crack depth. The crack occurred at the mid-span. The crack depth is increased from 0% to 50% of the thickness. Other parameters are the same as those of Case 1 in Section 3.2.2. Figure 9 shows the pattern change of the first eigenvalue induced by the growing crack depth. The figure below is an enlarged view of the area in the red box of the figure above. From Figure 9, the results show that changes in the crack depth mainly affect the distance between two adjacent peaks in the first eigenvalue curve. When the damage occurs, the distance between two adjacent peaks is increased with the crack depth.

#### 3.2.4. Orthogonality

MPCA can decompose the data into different coordinate axes in which each axis is orthogonal with other axes. For bridge SHM, the changes induced by structural damage need to be orthogonal with changes induced by other factors, such as the vehicle’s mass, the temperature, the road surface roughness, etc. In this section, the orthogonality between the vehicle’s mass and the damage is studied. As listed in Table 4, six cases have been studied. The parameters of Cases 1, 2, and 3 are the same as those in Section 3.2.2. Except for the crack depth, other parameters for Cases 4, 5, and 6 are the same as those in Section 3.2.2. Table 4 shows all six simulated scenarios.

**Figure 10:**
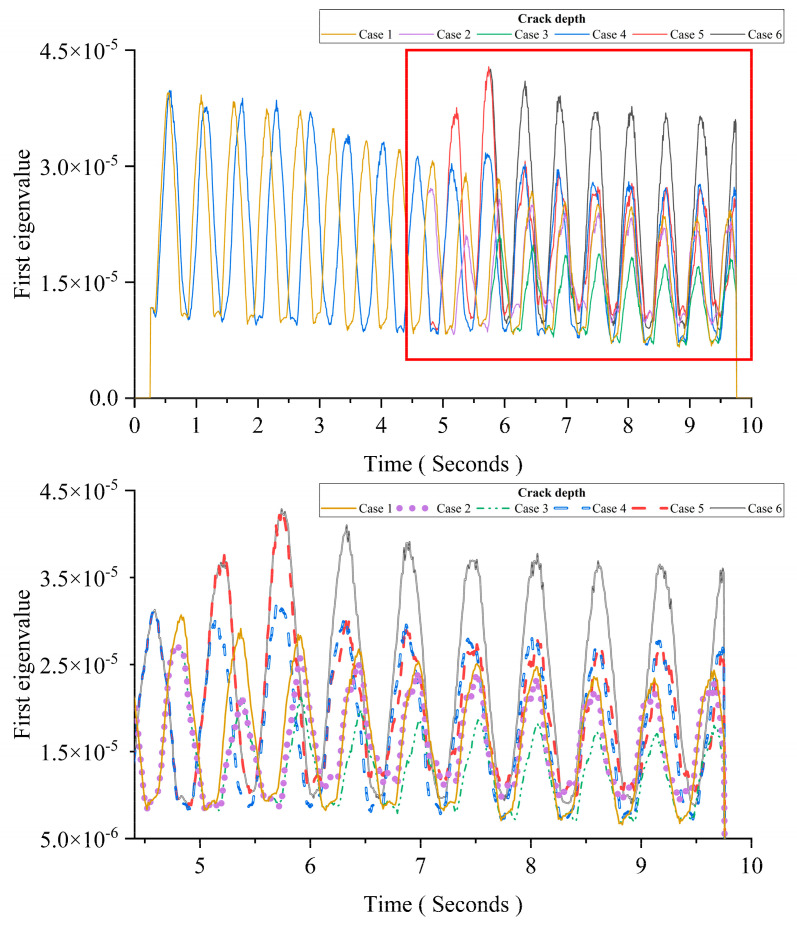
Result of six cases for the orthogonality.

Figure 10 shows results of six cases. The figure below is an enlarged view of the area in the red box of the figure above. Comparing Cases 1 to 3 with Cases 4 to 6, the results show that the influence of the damage is orthogonal with the influence of the moving mass. The influence of the damage is embodied in the distance between two adjacent peaks. Cases 4 to 6 show similar patterns, which are different from the intact beam’s patterns in Cases 1 to 3. The influence of the moving load’s mass is embodied in the magnitude of each peak. The first eigenvalue represents the amount of information at the corresponding time in acceleration responses. For the intact beam, the increase in the moving mass leads to the decrease in the first eigenvalue. For the damaged beam, the increase in the moving mass leads to the increase in the first eigenvalue. For Cases 1, 2, 4, and 5, the peak magnitude after 6 s is the same, and the results shows that there is a correlation between the peak magnitude and the moving mass. It could be used for estimating the moving mass.

#### 3.2.5. Temperature Influence

This section aims to investigate the temperature effect using MPCA. As listed in Table 5, there are six cases. Two scenarios are simulated: the change in the uniform temperature of the whole beam and the change in the temperature gradient at the cross-section. The temperature gradient is slightly changed when the vehicle is passing the bridge. All of the considered temperature changes are linear, and the moving vehicle passes the bridge in a short time period. There is no damage for Cases 1, 3, or 5, and the crack depth is 50% of the height for Cases 2, 4, and 6. The uniform temperature difference is used to simulate the temperature difference between day and night in mid-summer. The temperature gradient is used to simulate it under strong sunlight conditions. The other parameters are the same as in Case 1 in Section 3.2.4.

**Figure 11:**
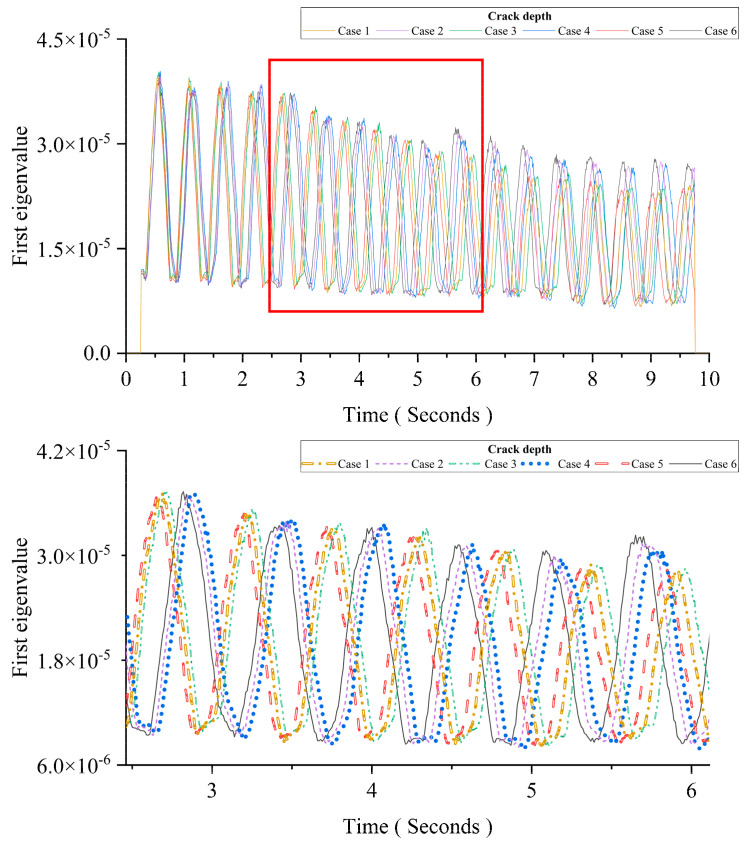
Temperature’s impact on MPCA.

Figure 11 shows the first eigenvalues of MPCA for different cases. The figure below is an enlarged view of the area in the red box of the figure above. From the figure, the first eigenvalue curve is changed with both the uniform temperature and the temperature gradient. Compared the results of Cases 1, 3, and 5, the time interval between two adjacent peaks for a case is increased with the uniform temperature, and the amplitude of the first eigenvalue curve is increased with the temperature gradient. Comparing the results of Cases 1, 3, and 5 and those of Cases 2, 4, and 6 shows that the deviation caused by the temperature change is concentrated in a specific, small range, and the change induced by the damage is much larger with a curve pattern.

### 3.3. Damage-Sensitive Features

#### 3.3.1. Observation

**Figure 12:**
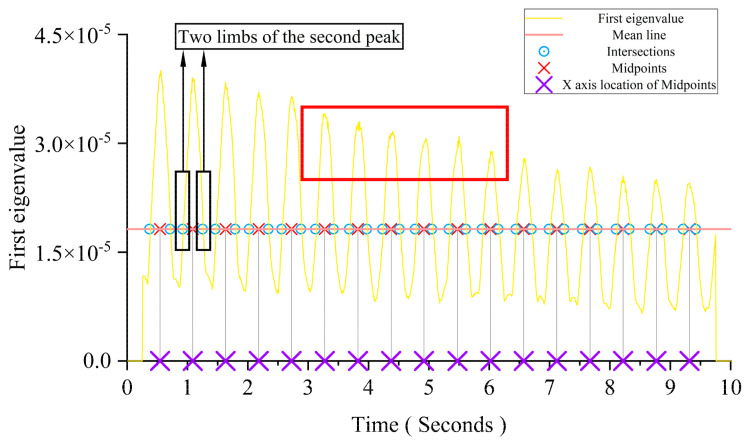
The details of the DSF’s construction.

According to previous discussions, the crack depth mainly affects the time interval between two adjacent peaks in the first eigenvalue curve, and the time interval is increased with the crack depth. The first eigenvalue curve has an obvious and identifiable pattern. There is some sawtooth interference information around the peak. The damage detection accuracy will be affected by these sawtooth liked oscillations. To avoid the influence of these perturbation, a smoothing treatment is adopted. Following the PCA, the first eigenvalue curve of the principal component is needed. Figure 12 shows that the steadiest part that can reflect the main trend of the first eigenvalue is the two limbs of each peak. Thus, the mean line of the first eigenvalue curve is taken from the first eigenvalue curve. The midpoints of each pair of intersections in each peak are taken as the foundation of the DSF’s construction.

#### 3.3.2. Construction

**Figure 13:**
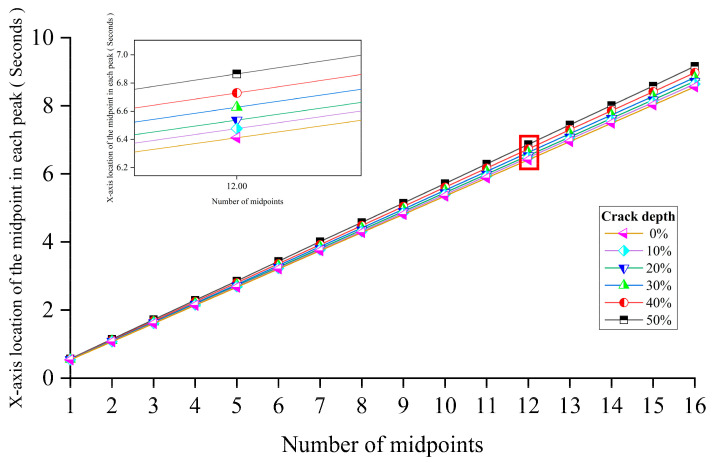
The growth trend of each peak’s midpoints.

Figure 13 shows that the growth trend of the *x*-axis location of each midpoint is linear. The thumbnail in this figure is an enlarged view of the red boxed area in Figure 12. The crack depth’s change will influence the inclination of each midpoint’s *x*-axis location’s growth trend line. In this section, all cracks occurred at the mid-span.

**Figure 14:**
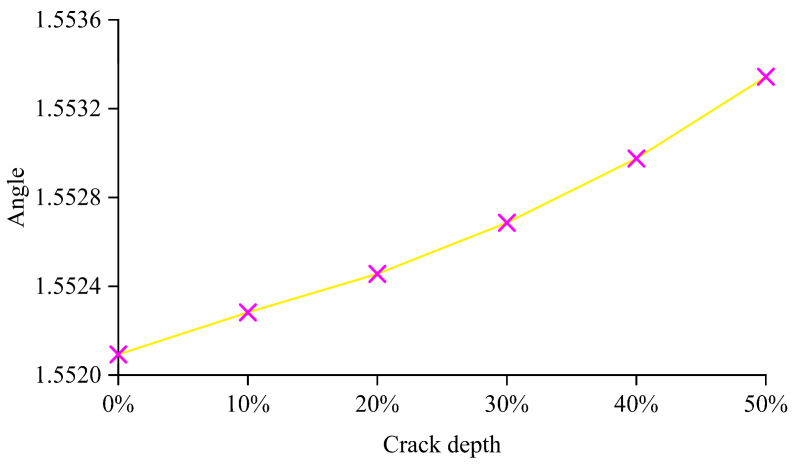
The angle of each line when the crack is growing.

Thus, the gradient of the line in Figure 13 is used as the DSF. The numerical derivatives of each pair of discrete midpoints are obtained. The mean of all numerical derivatives in each line is calculated corresponding to its crack depth. The angle of each line is obtained by the mean’s arctangent. Figure 14 shows the gradient of each line, which refers as the DSF, is growing corresponding to its crack depth’s growth.

#### 3.3.3. Influence of the Crack’s Location

**Figure 15:**
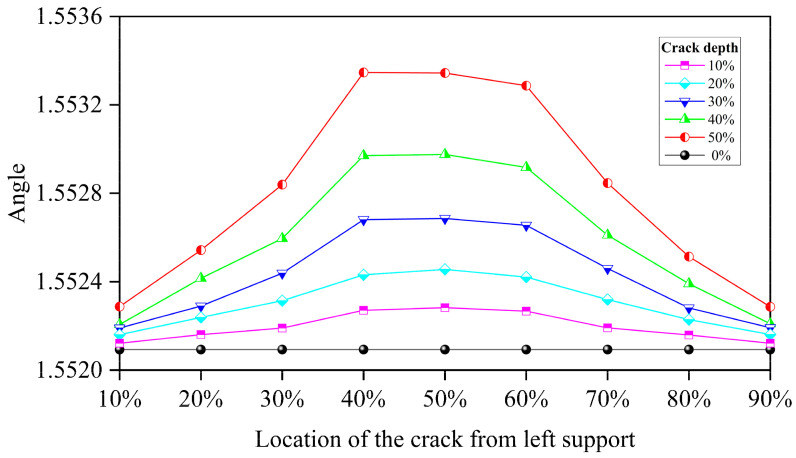
The angle of each line when the crack is located at different positions.

Figure 15 shows the crack location’s influence. The results reveal that for each crack depth, the crack located around the midpoint of the beam has a larger influence than the crack located near the beam’s end. This phenomenon will be more obvious when the crack grows deeper. This result complies with the beam’s dynamic analysis theory. In PCA, the first eigenvalue represents the variance in the first dimension. In this study, the first dimension is dominated by the acceleration changes caused by the moving loads. The vertical fluctuation and slow decline of the first eigenvalue reveal that the amount of the information (energy) brought by the moving load is gradually dissipated in the beam’s response over time due to the beam’s vibration. The existence of cracks will change the rate of this dissipation. Therefore, the increases in the distance between each midpoint represent these crack-induced changes. Additionally, this DSF reflects the damage of the beam from the overall perspective, which is the so-called equivalent crack depth. Because cracks in actual structures are distributed near the damaged area of the beam, this DSF can provide a more realistic beam damage situation. When the crack depth is greater than 30%, the Maxwell–Betti reciprocal theorem is no longer valid due to the nonlinearity caused by the cracks. Thus, although the two damage locations with a distance of 1/10 L on both sides of the beam’s midpoint are symmetrical in space, the damage extent reflected by these two DSFs is no longer consistent due to the directionality of the moving load on the time axis. In this case, the first eigenvalue is dominated by the moving load and the breathing crack. Within this range, the moving load passing through the crack earlier means that the beam has more time to dissipate, so the reflected degree of the damage will be slightly higher than in the other places where the moving load passes the crack later. Therefore, the proposed DSF describes the damage extent of the beam from a dynamic perspective. In other words, it depicts the beam’s “rhythm”. The traditional modal analysis describes the beam’s vibration from a static perspective. The magnitude of the first eigenvalue represents a measure of the maximum variance direction’s dimension. As the simply supported beam, the direction of the maximum variance of the measuring points’ acceleration is in the direction of the gravity axis when a uniaxial moving load is passing. Therefore, MPCA can capture a continuous peak fluctuation of the same magnitude in the first eigenvalue curve.

## 4. Experimental Investigation

A laboratory study has been conducted in this study. The experimental data from Zhu and Law [40] have been used. The first eigenvalue curve has been obtained using the proposed method in Section 3. The Gaussian window is adopted to reduce the measurement noise and the vehicle–bridge interaction. The selection of the window parameters is also discussed. The results of the obtained damage-sensitive feature based on these laboratory data using the proposed method in Section 3 are presented and discussed.

### 4.1. Experimental Setup

**Figure 16:**
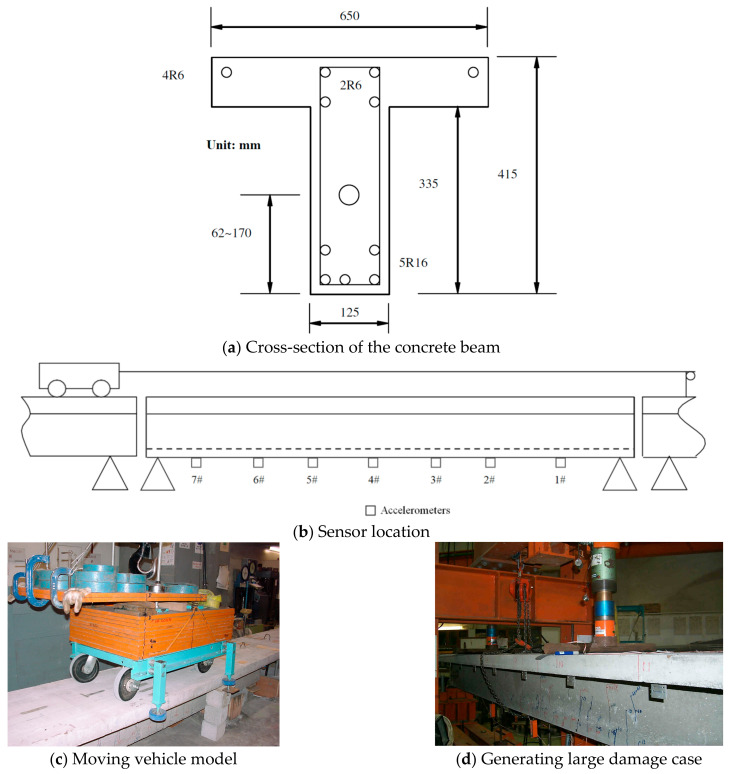
Experimental setup.

Figure 16 shows the experimental setup. The cross-section of the concrete beam is shown in Figure 16a. As shown in Figure 16b, the whole experimental beam is composed of three T-section reinforcement concrete beams: the front beam, the main beam, and the tail beam. The front and tail beams are 4.5 m long each. The main beam is 5.0 m long. The gaps between these three beams are 10 mm. An electric motor is used to pull the vehicle along the beam at a speed of approximately 0.5 m/s. The vehicle’s axle spacing is 0.8 m, and its wheel spacing is 0.39 m. There are two vehicle models with different weights used in this study. The whole weight of the first vehicle model (without an elastic spring) is 10.60 kN, with a front axle load of 5.58 kN and a rear axle load of 5.02 kN. The whole weight of the second vehicle model (with an elastic spring) is 15.00 kN, with a front axle load of 6.20 kN and a rear axle load of 9.00 kN. Because the mass of the whole concrete beam is 1050 kg, the weight ratios between the vehicle and the beam bridge for these two vehicle models are 1.01 and 1.43, respectively. Figure 16c,d include the photos taken during this experiment. Figure 16c shows the vehicle model passing through the beam, and Figure 16d shows the large damage case being generated.

As shown in Figure 16b, seven accelerometers are evenly installed along the beam at the bottom surface. Thirteen photo-electric sensors are distributed on the lead and the main beams with 0.56 m spacing to measure the vehicle’s moving velocity. The third and thirteenth photo-electric sensors are installed at the entry and exit points of the main beam, respectively. The INV300E data acquisition system is used to obtain the response data. The duration of each test is 30 s, and the sampling frequency is 2024.292 Hz.

**Figure 17:**
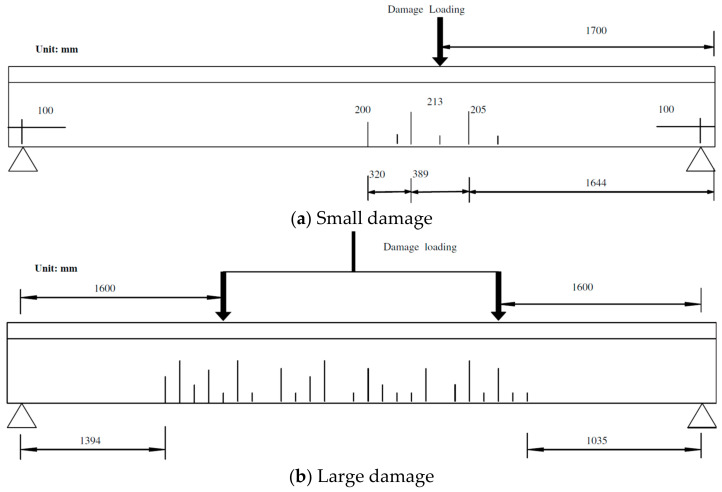
The damage loading and the crack zone.

A three-point load system is used to create the damage. The small damage is created by deploying the load at 1/3 L from the beam’s right support, as marked in Figure 17a. The load is gradually added in 2 kN increments. Several tensile cracks obviously appear on the beam rib when the load reaches 36 kN. When the load is 50 kN, the largest crack at the beam’s bottom is measured, and it has a 0.10 mm width. This crack is located close to the loading point but on the span inside, with a 213 mm depth and a 760 mm wide crack zone visually. The beam is unloaded after the load is kept for 30 min. Then, the crack at the beam’s bottom decreases to a 0.025 mm width and partly closes. These descriptions are referred to as the small damage case.

For the large damage case, a 50 kN load is first loaded at 2/3 L of the beam from the right support using the three-point load system. This produces a crack pattern similar in extent and magnitude to the existing crack zone at 1/3 L. After that, a four-point load system is used for further loading, as marked in Figure 17b. The final total load is 105 kN without the main reinforcement yielding, and the largest crack is located near the beam’s midpoint, with a 281 mm depth. This crack has a 0.1 mm width at the beam’s bottom when the load is 105 kN. When the beam is unloaded after keeping the 105 kN static load on the top surface for 30 min, this crack’s width reduces to 0.038 mm. The crack zone is 2371 mm long.

### 4.2. The Gaussian Window

**Figure 18:**
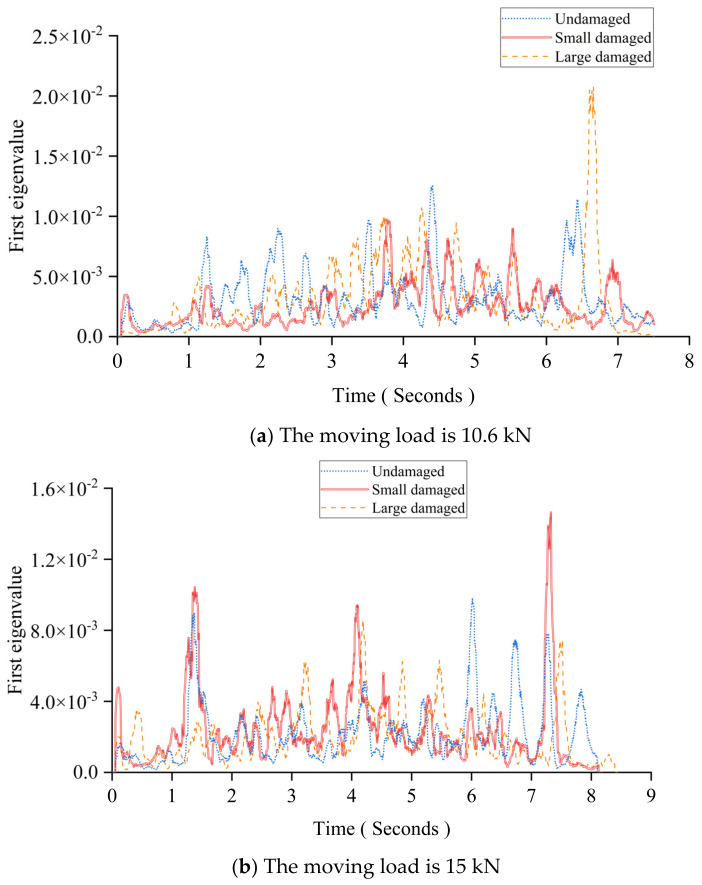
The first eigenvalue curve in this experimental study.

Figure 18 shows the first eigenvalue curves under different moving vehicle models. Figure 18a shows the results for the undamaged beam and the beams with the small and large damage under a 10.6 kN moving vehicle. Figure 18b shows the results under a 15 kN moving vehicle. The results show that the pattern of the first eigenvalue curve is severely affected due to the existence of measurement noise and the vehicle–bridge interaction. Measurement noise and the vehicle–bridge interaction are two main influencing factors for the first eigenvalue curve. Upon comparing Figure 18a with Figure 18b, because the latter uses a 30% heavier vehicle, it can be seen that its curve is less affected by those influencing factors. The window length in these two cases is randomly selected, with 54 sampling intervals for comparison and illustration. Therefore, following this idea, if a smaller window can be used, more components caused by the moving load can be extracted. However, due to the limitation of the PCA algorithm, the window’s length cannot be less than the number of input signal channels. Additionally, a small window length may lose the important information created by the moving loads. Thus, the Gaussian window is proposed.

**Figure 19:**
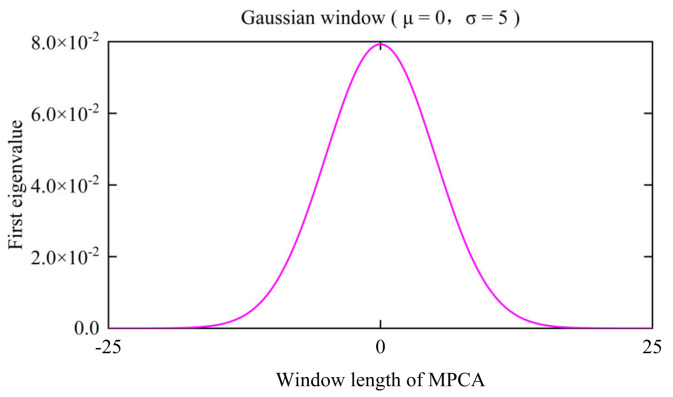
The Gaussian window.

The Gaussian window draws on the idea of regularisation. Figure 19 shows a Gaussian window (σ (0, 5)) when the normal window’s length is 50 sampling intervals. It is equivalent to adding a penalty term: the farther the time is from the current moment, the smaller the impact on the current moment. The greater the weight in the middle, the deeper the consideration of instantaneous effects. The influencing factors’ impact on the time axis is diffuse, so their effects can be significantly reduced by the Gaussian window, and the proportion of the vehicle excitation in this window is magnified at the same time. In other words, PCA is a multi-channel data processing method, and the additional normal moving window expands the eigenvalue along the time axis. Through the expansion of eigenvalues, the same overall movement trend of each measuring point on the bridge is displayed along the time axis, and each interference factor is expanded on the time axis. Therefore, the Gaussian window realigns this expansion on the time axis again. This minimises the effect of interference factors on the detection results.

**Figure 20:**
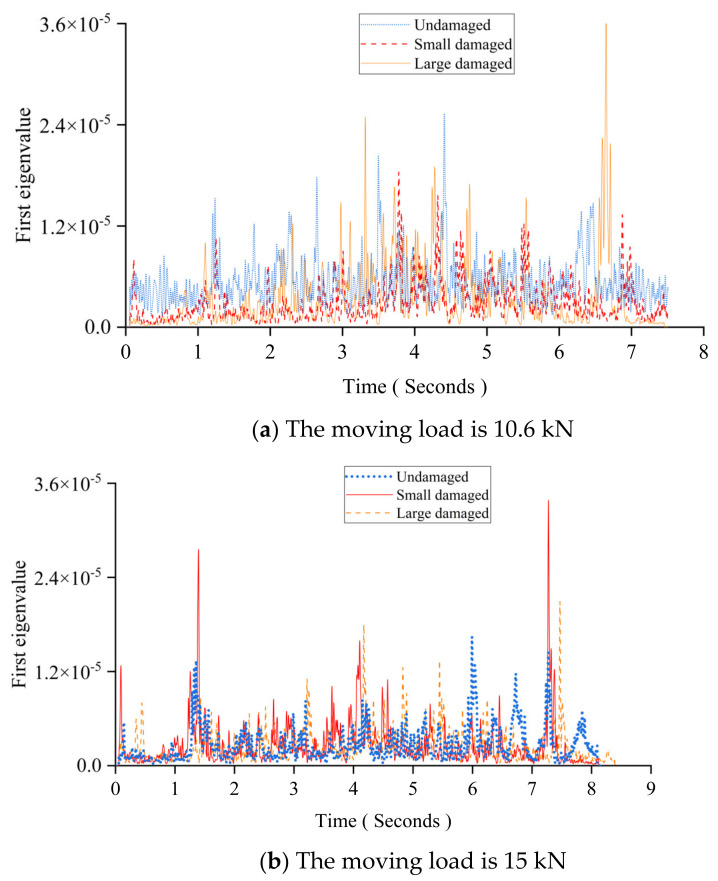
The first eigenvalue curve with the Gaussian window in this experimental study.

In Figure 18, the first eigenvalue curve has many distortions or buckling caused by the noise, the vehicle–bridge interaction, or other factors, like human interference or operational error. Figure 20 shows the first eigenvalue curves smoothed by the Gaussian window. The Gaussian window emphasizes the data closer to the current time by giving more coefficient weight to reduce the effect from those influence factors on the first eigenvalue curve.

### 4.3. Parametric Study

#### 4.3.1. The Effect of the Window Length

**Figure 21:**
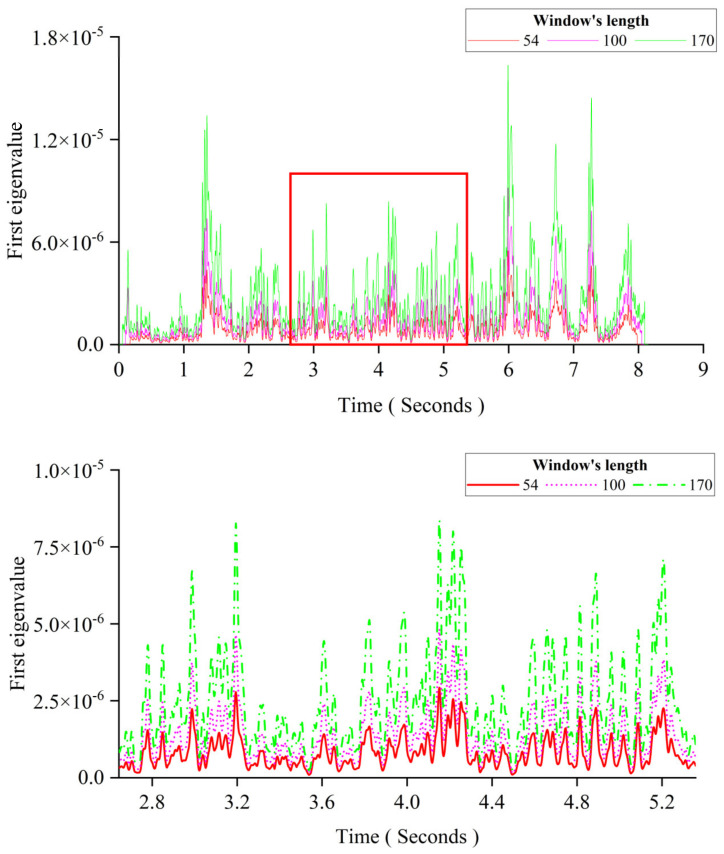
The effect of the Gaussian window’s length.

This section will discuss the selection of the window’s parameters. The first step is to investigate the influence of the window length on the first eigenvalue curve. In this section, the responses of the undamaged experimental beam under the 10.6 kN moving load are used. Figure 21 shows the first eigenvalue curves obtained under three commonly used lengths of the window. The image below is an enlarged view of the area in the red box of the image above. It is not recommended to use a length more than 200 times the sampling interval because a large window will greatly increase the MPCA’s computational cost. From the first eigenvalue curves under different window lengths, we can see they have the same shape. Their only difference is the magnitude. The window length will not influence the damage detection because the damage influences the distance between each pair of adjacent peaks instead of the magnitude. Therefore, the second contribution of the Gaussian window is that it simplifies the problem of the window length’s selection. The Gaussian window transforms the selection of the window length into the hyperparameter σ’s selection of itself and allows for the use of a small window to reduce the amount of calculation.

#### 4.3.2. Hyperparameter of the Gaussian Window

The choice of the hyperparameter σ will affect the Gaussian window’s attention degree to the current moment and the tolerance of the influencing factors. If the value is too small, the anticipated effect cannot be achieved. If the value is too large, all of the information carried in the data will be destroyed (by only taking the value at the current time *t* into account). The numerical model is used to find the optimal value. The parameters of the simulated beam are the same as in Case 1 in Section 3.2.2, and the length for both normal and Gaussian windows is 50 sampling intervals.

**Figure 22:**
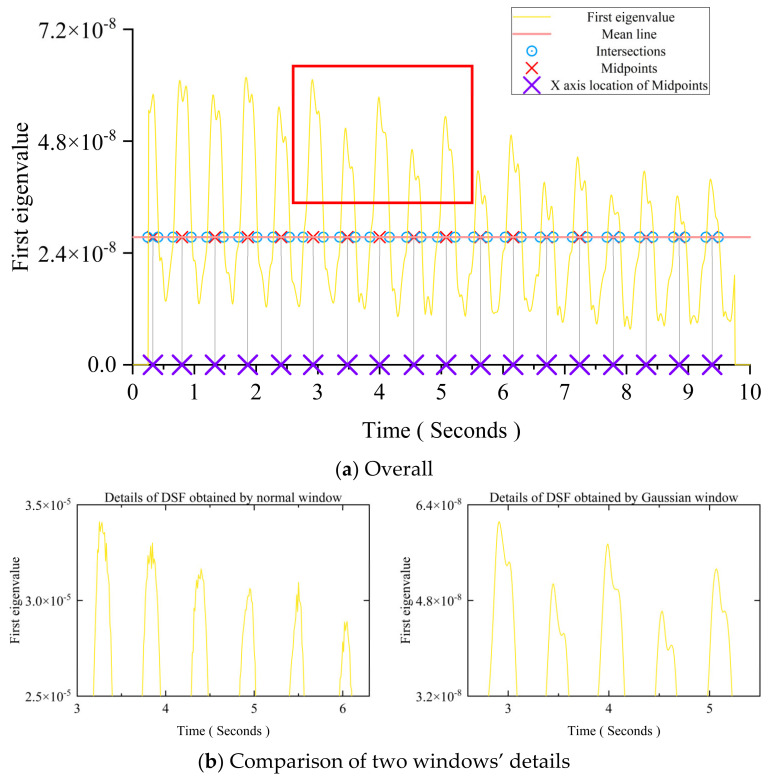

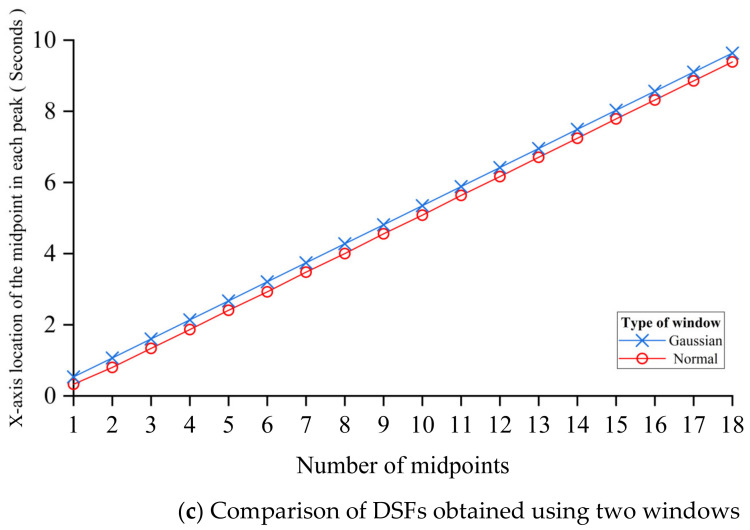
The results of numerical studies using two types of windows.

Figure 22a shows the first eigenvalue curve processed using the Gaussian window (σ (0,5)). Compared with the curves in Figure 12, the curve obtained using the Gaussian window is much smoother. Figure 22b shows the enlarged view of the area in the red box of Figure 12 and Figure 22a, correspondingly. Figure 22b reveals that the Gaussian window can erase the distortions near the peak and trough areas of the first eigenvalue curve. Except for the window’s type, the damage conditions and other parameters for obtaining the DSFs in Figure 12 and Figure 22a are the same. Figure 22c shows that the DSFs obtained using the normal window and the Gaussian window are almost the same. They both have the same gradient, which could be an indicator of the beam’s damage extent. Thus, the σ (0,5) can be used as the optimal hyperparameter of this study. The Gaussian window (σ (0,5)) can smooth the first eigenvalue curve without affecting the accuracy of the DSF for eliminating the influence of interference factors.

### 4.4. Experimental Results and Discussions

**Figure 23:**
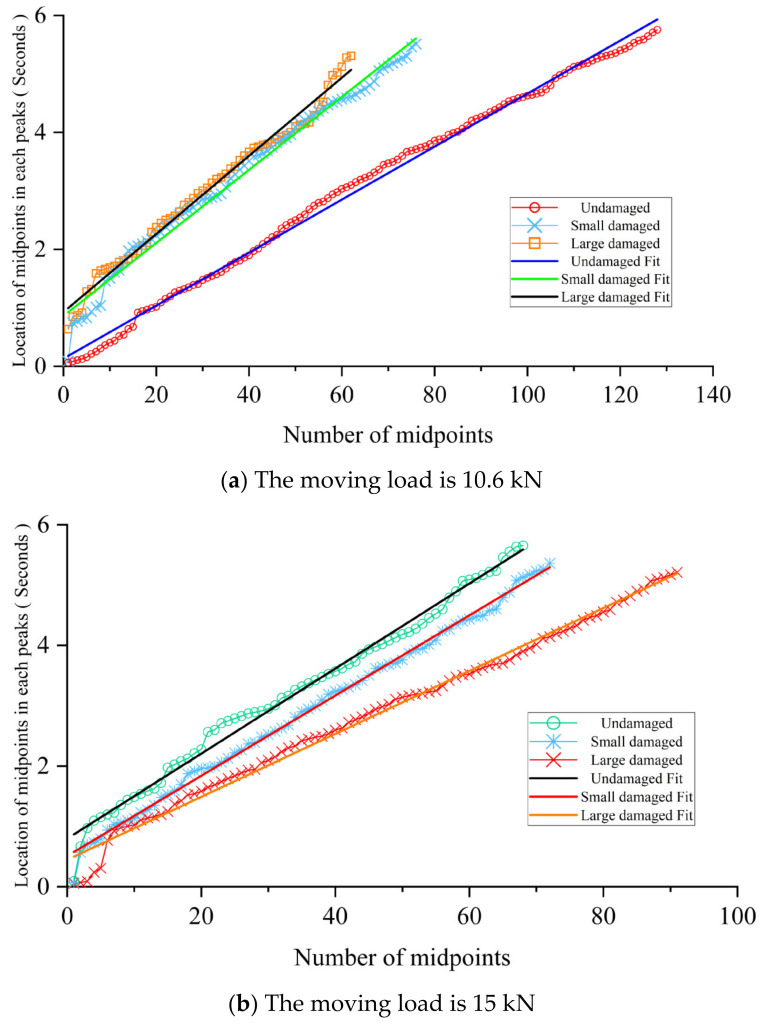
The results of the proposed DSF.

Figure 23 shows that the proposed DSF can distinguish the beam’s damage extent well when using the experimental dataset. Because the heavier vehicle can better excite the response due to the crack, the result in Figure 23b is better than that in Figure 23a in distinguishing the damage extent. Due to the existence of the elastic spring, the wheel can better maintain contact with the concrete surface. The result in Figure 23b is smoother and more continuous than that in Figure 23a. The size of the gradient reflects the “rhythm” of the beam. The cracks weaken the effective cross-sectional area of the beam, thereby hindering the transmission of information in the beam. In this case, the speed at which the first eigenvalue reaches each local extreme value will slow down, and the gradient will become larger. Due to the existence of the elastic spring, part of the energy in the response is transferred in the beam using the vehicle as the transmission path. This leads to an increase in the information transmission bandwidth of the beam. When a visible crack zone occurs in the beam, the load in this area will be borne more by the steel bars. Because the vehicle in Figure 23b is 30% heavier than the one in Figure 23a, this phenomenon will become deeper as the load increases. Because the load carried by the steel bars increases as the cracks deepen, the overall “rhythm” of the beam becomes faster, and the gradient becomes smaller. Thus, these two points will reverse the change pattern of the DSF when the cracks grow, but this requires further study.

## 5. Conclusions

A new structural damage detection method based on moving principal component analysis (MPCA) has been developed for condition assessment of a highway bridge under moving vehicles. Numerical and experimental results show that the proposed method is effective and accurate in detecting structural damage. The following points are concluded in this paper:

(1) The gradient of the first eigenvalue curve obtained from raw acceleration signals using MPCA is used as the damage-sensitive feature (DSF) of the highway bridge. The DSF can clearly reflect the existence of the breathing crack on the bridge. The change pattern of the first eigenvalue curve induced by the different vehicle’s mass, temperature fluctuations, different damage depths, and locations has been studied, and the results show the robustness, accuracy, and practicality of the proposed DSF.
(2) The DSF is not limited to a few pre-considered parameters but rather reflects the beam’s damage extent from a dynamic perspective. As the damage in the concrete structures is a crack zone in the actual situation, the equivalent crack depth indicated by this DSF could reflect the damage extent of the beam.
(3) The experimental results show that the Gaussian window is useful for improving the performance of MPCA on actual datasets. This window can filter out the impact of effects like measurement noise and the vehicle–bridge interaction. The experimental results also show that the DSF can detect and distinguish crack damage of different extents under the different vehicles’ weights.
(4) The method has been verified with a bridge subjected to one moving vehicle. Further studies are needed for practical applications, with a bridge subjected to multiple vehicles.

## Figures and Tables

**Table 1:** The temperature coefficients of the beam’s parameters [16].

Parameters	Formula	Coefficient	$\mathrm{Steel}({/}^{\circ }C)$	$\mathrm{Concrete}({/}^{\circ }C)$
Expansion	$A=A_{0}(1+\alpha_{L}\Delta T{)}^{2}$	$\alpha_{L}$	$1.2\times 10^{-5}$	$1.3\times 10^{-5}$
Young’s modulus	$E=E_{0}(1+\alpha_{E}\Delta T)$	$\alpha_{E}$	$-3.2\times 10^{-4}$	$-7.2\times 10^{-3}$
Second moment of inertia	$I=I_{0}(1+\alpha_{L}\Delta T{)}^{4}$	$\alpha_{L}$	$1.2\times 10^{-5}$	$1.3\times 10^{-5}$

**Table 2:** The numerical model’s natural frequencies.

Natural Frequencies	
By Zhu and Law [40]	By the Proposed Method
0.94	0.9375
3.75	3.7501
8.44	8.4377
15.00	15.0004
23.44	23.4390
33.75	33.7547

**Table 3:** The three simulated cases.

Case	Mass Change	Time Duration	
		Start Time	End Time
1	0%	-	-
2	1%	5 s	6 s
3	1%	5 s	10 s

**Table 4:** Six cases for the orthogonality.

Case	Mass Change	Time Duration		Crack Depth
		Start Time	End Time	
1	0%	-	-	0
2	1%	5 s	6 s	0
3	1%	5 s	10 s	0
4	0%	-	-	50%
5	1%	5 s	6 s	50%
6	1%	5 s	10 s	50%

**Table 5:** Cases for the temperature effect using MPCA.

Case	Uniform Temperature (°C)	Temperature Gradient (°C)		Crack Depth
		Start Time	End Time	
1	25	0	0	0
2	25	0	0	50%
3	40	28	25	0
4	40	28	25	50%
5	10	20	15	0
6	10	20	15	50%

## Data Availability

Data are contained within the article.
